# Jasmonates Alleviate the Harm of High-Temperature Stress During Anthesis to Stigma Vitality of Photothermosensitive Genetic Male Sterile Rice Lines

**DOI:** 10.3389/fpls.2021.634959

**Published:** 2021-03-29

**Authors:** Jing Chen, Wenqian Miao, Keqi Fei, Hongli Shen, Yujiao Zhou, Yan Shen, Chaoqing Li, Jiang He, Kuanyu Zhu, Zhiqin Wang, Jianchang Yang

**Affiliations:** Jiangsu Key Laboratory of Crop Genetics and Physiology, Co-innovation Center for Modern Production Technology of Grain Crops, Yangzhou University, Yangzhou, China

**Keywords:** antioxidant ability, high-temperature stress, jasmonates, photo-thermo-sensitive genic male sterile line, rice (*Oryza sativa* L.), stigma vitality

## Abstract

Using photothermosensitive genic male sterile (PTSGMS) rice (*Oryza sativa* L.) lines to produce hybrids can obtain great heterosis. However, PTSGMS rice lines exhibit low stigma vitality when high-temperature (HT) stress happens during anthesis. Jasmonates (JAs) are novel phytohormones and play vital roles in mediating biotic and abiotic stresses. Little is known, however, if and how JAs could alleviate the harm of HT stress during anthesis to the stigma vitality of PTSGMS lines. This study investigated the question. Two PTSGMS lines and one restorer line of rice were pot-grown and subjected to normal temperature and HT stress during anthesis. The stigma exertion rate, sigma fresh weight, stigma area, contents of JAs, hydrogen peroxide (H_2_O_2_), and ascorbic acid (AsA), activity of catalase in stigmas, and the number of pollens germinated on the stigma of PTSGMS lines were determined. The results showed that a rice line with higher JAs content in the stigma under HT stress showed lower H_2_O_2_ content, higher AsA content and catalase activity in stigmas, larger stigma area, heavier stigma fresh weight, more pollens germinated on the stigma, and higher fertilization and seed-setting and rates. Applying methyl JAs during anthesis to rice panicles decreased the accumulation of reactive oxygen species and enhanced stigma vitality, thereby increasing fertilization and seed-setting rates of the hybrids of PTSGMS rice lines under HT stress. The results demonstrate that JAs attenuate the injury of HT stress to the stigma vitality of PTSGMS rice lines through enhancing antioxidant ability.

## Introduction

Using “two-line method” to produce hybrid rice (*Oryza sativa* L.), namely, a hybrid produced by a photothermosensitive genic male sterile (PTSGMS) line crossed with a restorer line, could produce great heterosis (Yang et al., 2003; Deng et al., 2013; Ma and Yuan, 2015; Yuan, 2017). However, PTSGMS rice lines are sensitive to temperature and usually exhibit serious reduction in fertilization ability when subjected to high-temperature (HT, ≥35°C) stress during anthesis (Mou, 2016; Yang et al., 2016; Chen et al., 2020, 2021).

With the increase in global greenhouse effect and changes in climate, HT stress has become one of the most detrimental stresses among constantly changing environmental factors (Jagadish et al., 2010; Luo and Lau, 2019; Karwa et al., 2020; Wahab et al., 2020). The flowering or anthesis stage in rice plants is most vulnerable to HT stress (Jagadish et al., 2010; Luo and Lau, 2019; Chen et al., 2020; Yang et al., 2020). For a conventional male fertile rice cultivar or a line, HT stress during anthesis usually causes failure in anther dehiscence, pollination, and germination of pollen grains, leading to a low seed-setting rate and a low grain yield (Jagadish et al., 2010; Mittler and Blumwald, 2010; Cao et al., 2015; Li et al., 2018; Jiang et al., 2020). In contrast, for a PTSGMS rice line that shows total male sterility when temperature is >23 or 24°C (Yuan, 1997; Ma and Yuan, 2015; Mou, 2016), HT stress during anthesis mainly impairs spikelet opening and reduces pistil activity and consequently, seriously decreases grain yield of a hybrid produced by the line crossed with a restorer line (Zhu, 2016; Chen et al., 2020, 2021; Yang et al., 2020).

Although few studies have recently investigated the effect of HT stress during anthesis on spikelet-opening and pistil (ovary) activity of PTSGMS rice lines (Chen et al., 2020, 2021; Yang et al., 2020), little information is available about if and how HT stress during anthesis influences stigma vitality of PTSGMS lines. Generally, stigma vitality of rice includes both morphological and physiological aspects, such as the stigma area, stigma exertion, antioxidant ability, and hormonal levels (Yin et al., 2014; Costa et al., 2016; Chen et al., 2020). A larger stigma area is considered as one of the most important ancillary features reinforcing the function of this reproductive system (Dulberger, 1992; Costa et al., 2016) and could maintain a higher rate of pollination for a male sterile rice line by receiving more fertile pollens (Li and Johnston, 2001; Marathi and Jena, 2014; Costa et al., 2016). While with the increase in air temperature, HT stress during anthesis often causes desiccated stigma and stigma burn among some tomato genotypes (Prasad and Djanaguiraman, 2014). Those desiccated stigma or stigma burn usually lead to decreased stigma area. As one of the key factors for improving the outcrossing ability of PTSGMS rice lines, stigma exertion is an important character of stigma vitality because exerted stigmas remaining outside the glumes after spikelet-opening often receive more pollens than those with hidden stigmas remaining inside the glumes after flowering, and a higher stigma exertion rate could have higher spikelet fertility in rice (Xu and Shen, 1988; Yin et al., 2014). Under heat stress, the stigma exertion rate was observed to be decreased due mainly to the decrease in stigma vitality, but the decrease varied with genotypes and the temperature treatment period (Fernandez-Munoz and Cuartero, 1991; Ramesh et al., 2017; Chen et al., 2020, 2021).

Usually, HT stress could produce more reactive oxygen species (ROS), such as hydrogen peroxide (H_2_O_2_), and reduce antioxidant ability, such as decreases in catalase activity and ascorbic acid (AsA) content, in plant organs (Galen and Plowright, 1987; Dafni and Maués, 1998; Prasad and Djanaguiraman, 2011, 2014; Snider et al., 2011; Yang et al., 2020). It is generally proposed that phytohormones are signaling molecules and play vital roles not only in regulating plant growth and development, but also in mediating the physiological processes responding to a variety of biotic and abiotic stresses including HT stress (Yang et al., 2001; Zhang et al., 2009; Wani et al., 2016; Zhou et al., 2020). Among them, jasmonates (JAs), including jasmonic acid (JA) and methyl jasmonate (MeJA), have attracted great attention because they have been observed to minimize the damage of HT stress to plants in recent studies (Nguyen et al., 2019; Chen et al., 2020; Yang et al., 2020). It has been observed that JAs could confer thermotolerance by enhancing the expression of genes in the JA biosynthesis and signal pathways in *Arabidopsis* (Clarke et al., 2009; Kazan, 2015). Increases in JAs contents in rice lodicules by applying JA or MeJA have been reported to significantly increase opened-spikelet rate subjected to HT stress during anthesis (Yang et al., 2020). The physiological mechanism by which JAs ameliorate tolerance to abiotic stresses is mainly explained to maintain the redox homeostasis in plants including *Arabidopsis*, rice, wheat, and tomato (Hasanuzzaman et al., 2013; Farhangi-Abriz and Ghassemi-Golezani, 2019; Chen et al., 2020; Yang et al., 2020). However, little is known whether JAs could alleviate the effect of HT stress during anthesis on the stigma vitality of PTSGMS rice lines through regulating ROS and antioxidant system (AOS) in the stigma.

The objective of this study was to test the hypothesis that JAs may mediate the effect of HT during anthesis on stigma vitality of PTSGMS rice lines by regulating ROS and AOS levels. The contents of JA and MeJA, the major forms of JAs in rice plants, were determined. The morphological and physiological traits reflecting stigma vitality, that is, the area, exertion, and fresh weight of stigmas, pollen germination on the stigma, H_2_O_2_ and AsA contents, and catalase activity in the stigmas, were measured. The effect of applying MeJA on stigma vitality was investigated to verify the role of JAs in enhancing tolerance of PTSGMS lines to HT stress.

## Materials and Methods

### Plant Materials and HT Treatment

Experiments were conducted at the research farm of Yangzhou University, Jiangsu Province, China (32°30′ N, 119°25′ E, 21 m altitude) during the rice-growing season (May–October) in 2017, 2018, and 2019. Two PTSGMS rice (*O. sativa* L.) lines currently used in China because of their greater heterosis than other PTSGMS rice lines (Ma and Yuan, 2015; Mou, 2016; Yuan, 2017), Peiai 64S, Shen 08S, were used. A restorer line Yangdao 6 (a conventional rice variety) was used as a normal pollen donor). The three test lines were pot-grown, and the growth conditions were described previously (Chen et al., 2020, 2021; Yang et al., 2020).

When ~50% of the plants were heading from the sheaths of flag leaves, the plants heading on the same day were chosen and tagged from 50 pots of each line and each treatment and then were moved into four phytotrons (AGC-MR, Zhejiang Qiushi Environment Co., Ltd, Zhejiang, China) for both normal temperature (NT) and HT treatments with three rice lines in each phytotron. Treatment details were described by Chen et al. (2020, 2021). Briefly, during the period of 04:01–06:00, 06:01–08:00, 08:01–10:00, 10:01–14:00, 14:01–16:00, 16:01–18:00, 18:01–20:00, and 21:00–04:00 h, the temperatures (in °C) were 24, 26, 29, 32, 30, 28, 26, and 24, respectively, for the NT treatment (control), in accordance with the diurnal variation of air temperature at local weather conditions, and were 27, 30, 35, 39, 35, 32, 30, and 26, respectively, for the HT treatment. The light densities for both NT and HT were 200, 800, 1,000, 1,000, 1,000, 800, 100, and 0 μmol m^−2^ s^−1^, respectively, for the period mentioned previously. The relative humidity and CO_2_ concentration were kept at 70 ± 10% and 370 ± 20 μmol mol^−1^ for both NT and HT during the treatment, respectively, which were similar to outdoor conditions. The period of temperature treatment lasted for 48 h from 04:01 h on the first day to the 04:00 h on the third day of the treatment. During anthesis, panicles of both PTSGMS lines under either NT or HT were fertilized with the pollens from the restorer line under NT with the help of manual pollination. Each PTSGMS line for the fertilization had 20 pots as replications under either NT or HT.

### Sampling and Determination

Four hundred panicles showing spikelet opening were tagged from 30 pots of each rice line and each temperature treatment. The spikelets that opened at 9:00 h on the first day during treatment were sampled at 12:00 and 14:00 h on the first day during treatment, 14:00 h on the second day during treatment, and 14:00 h on the first day after treatment (the third day from the treatment), respectively, for the measurements of stigma area; stigma fresh weight; stigma exertion rate; the contents of JA, MeJA, H_2_O_2_, and AsA; and catalase activity in stigmas. Each physiological measurement had three biological replications.

### Stigma Exertion, Pollen Germination, and Stigma Area

The opened spikelets with an angle ≥10° between the palea and lemma showing the exertion of single stigma and/or dual stigmas on the panicle that headed out from the sheath of flag leaf were marked and counted at every 30 min from 07:00 to 15:00 until the spikelets on each tagged panicle were fully opened. The total number of opened spikelets and spikelets with single exerted stigma and/or with dual exerted stigmas from 18 tagged panicles were counted and recorded from the first day of temperature treatment to the finish of the whole heading period, with six panicles as one replication. The percentage of single stigma exertion, dual stigma exertion, and total stigma exertion on a panicle were calculated using the following formulas:

(1) $$\begin{array}{r} \text{Exertion percentage of single stigma }\left(\text{\%}\right)=\text{the number of}\\ \text{ opened spikelets with single stigma exertion}/\text{the total}\\ \text{ number of opened spikelets }\times 100 \end{array}$$

(2) $$\begin{array}{r} \text{Exertion percentage of dual stigmas }\left(\text{\%}\right)=\text{the number of}\\ \text{ opened spikelets with dual stigma exertion}/\text{the total}\\ \text{ number of opened spikelets }\times 100 \end{array}$$

(3) $$\begin{array}{r} \text{Exertion percentage of total stigmas }\left(\text{\%}\right)=\text{the number of}\\ \text{ opened spikelets with single and dual stigma exertion}/\text{the}\\ \text{ total number of opened spikelets }\times 100 \end{array}$$

For each PTSGMS line, 30 tagged spikelets that opened on the top of panicle at 9:00 h on the second day during treatment were fully fertilized with the pollens from the restorer line and then were sampled from each treatment. After 30 min of fertilization, 10 pollinated stigmas from each treatment were quickly taken out using tweezers and put into the precooled formaldehyde–acetic acid–ethanol fixative solution for 24 h, and then the pretreated samples were reserved in 70% (vol/vol) alcohol. Before the observation of pollen germination, the samples were put in 50% (vol/vol), 30% (vol/vol), and 10% (vol/vol) alcohol and distilled water, respectively, and rehydrated for 10 min each time. Ten M NaOH solution at 56°C was used to soften the samples after rehydration for about 10 min, and 0.1% (vol/vol) aniline blue dyeing solution was also used to stain the samples for 12–24 h. The stained samples were washed with distilled water twice and then put on a glass slide. After adding a drop of glycerin and covering a cover slip, the samples on the slide were used to observe the number of pollens germinated on the stigmas under a fluorescent microscope and used the image editing software (ZEN 2012, Carl Zeiss Microscopy GmbH, Zeiss, Germany) for calculation. The stigma area was also observed using a fluorescent microscope with the help of image editing software ZEN 2012.

### Quantification of JAs, ROS, and AOS in Stigmas

The extraction and quantification of JA and MeJA in the stigma were performed according to the methods of Liu et al. (2010) and Yang et al. (2020). The content of H_2_O_2_ in the stigma was determined using the method of Avramova et al. (2015). The AsA content and catalase activity in stigmas were assayed according to the protocols of Wang et al. (2013) and Gupta et al. (2016), respectively.

### Final Harvest and Statistical Analyses

The hybrids of PTSGMS lines crossed with the restorer line Yangdao 6 (Peiai 64S × Yangdao 6 and Shen 08S × Yangdao 6) from 10 pots for each treatment were harvested at maturity for the determination of grain yield and yield components as described previously (Chen et al., 2020, 2021; Yang et al., 2020). Analysis of variance was performed using the SAS/STAT statistical analysis package (version 9.12, SAS Institute, Cary, NC, USA), and correlation analysis was used to evaluate the relationships of JAs with other physiological and morphological traits of stigmas.

### MeJA Application

The experiment for MeJA application was conducted in 2019. Two PTSGMS lines of Peiai 64S and Shen 08S and one restorer line of Yangdao 6 were pot-grown and subjected to both NT and HT during anthesis as described above. At 09:30 to 10:00 of the first day during the temperature treatment, 100 μM MeJA (Sigma Chemical Co.) was sprayed to the panicles that headed out from the sheaths of flag leaves, with 4 mL per panicle. The MeJA concentration of 100 μM was chosen because such a concentration was observed to be an optimal dose for enhancing the tolerance of rice to HT stress (Yang et al., 2020). All the solutions contained ethanol at a final concentration of 0.05% (vol/vol). Control plants were applied with same dosage of deionized water containing the same concentration of ethanol. Each treatment had 35 pots as replications. Plants of 30 pots from each treatment were used for determining stigma fresh weight, stigma area, stigma exertion rate, contents of JA, MeJA, H_2_O_2_, and AsA, and activities of catalase in stigmas and the number of pollens germinated on single stigma. The measurements were made at 12:00 and 14:00 h on the first day during the temperature treatment, 14:00 h on the second day during the temperature treatment, and 14:00 h on the first day after the temperature treatment. Panicles of another five pots for each treatment were fertilized with the pollens from the restorer line Yangdao 6 for the determination of the seed-setting and fertilization rates and seed yield of the hybrids. The methods used for the determinations were the same as those described above.

To visualize the H_2_O_2_ accumulation in stigmas, ROS (mainly H_2_O_2_) accumulation in the stigmas and stigma receptivity were qualitatively analyzed as described by diaminobenzidine (DAB) stain (Lee et al., 2002) and LIVE green ROS detection staining methods (Prasad and Djanaguiraman, 2011; Djanaguiraman et al., 2017). Briefly, nine pairs of stigmas from each treatment at each sample time were quickly taken out from spikelets using tweezers and were vacuum-infiltrated with 0.1 mg mL^−3^ DAB in 50 mM Tris-acetate buffer, pH 5.0. Samples were incubated for 24 h at 25°C in the dark. To remove chlorophylls, the samples stained with DAB solution were transferred to 95% (vol/vol) ethanol for 3 times at every 30 min, after the samples were incubated at 25°C in the dark for 2 and 24 h, respectively. Accumulation of ROS in samples was then observed under a microscope. The Image-iT™ LIVE Green Reactive Oxygen Species Detection Kit (I36007) was used for quantifying the ROS accumulation in live cells and stigma receptivity according to Prasad and Djanaguiraman (2011) and Djanaguiraman et al. (2017), respectively.

## Results

### Levels of JAs, ROS, and AOS in Stigmas

**Figure 1:**
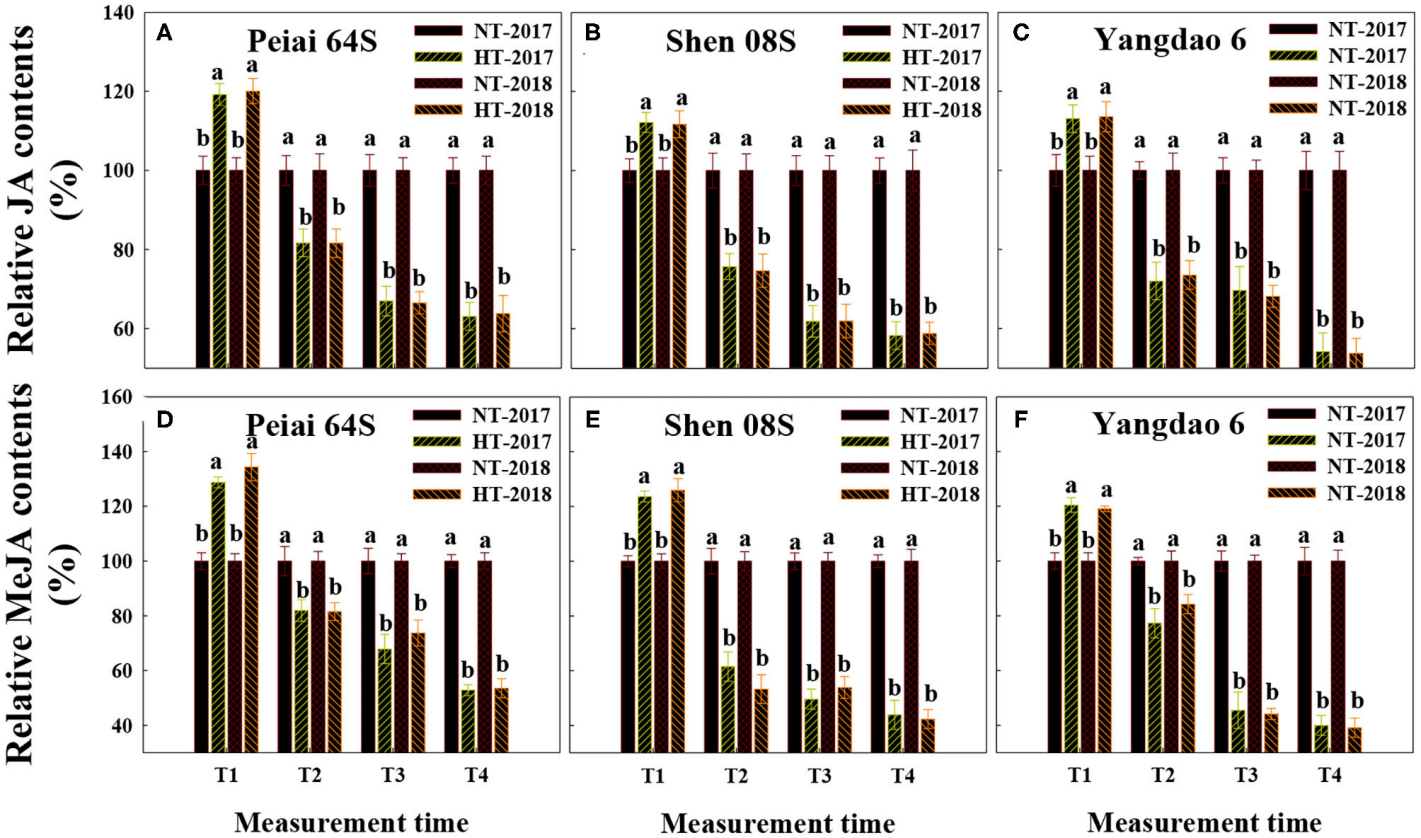
Effect of high-temperature stress during anthesis on the contents of jasmonic acid (JA) **(A–C)** and methyl jasmonate (MeJA) **(D–F)** in stigmas of rice. NT and HT represent normal temperature and high temperature, respectively, and the NT is taken as control. Data are expressed as relative values, and those of controls are taken as 100%. T1: 12:00 h on the first day during the temperature treatment; T2: 14:00 h on the first day during the temperature treatment; T3: 14:00 h on the second day during the temperature treatment; T4: 14:00 h on the first day after the temperature treatment. The bars are SD of three biological replications. Different letters above the bars represent significant differences at the *P* ≤ 0.05 level within the same rice line and the same measurement time.

Changes in the contents of JA and MeJA in the stigmas varied with measurement times and PTSGMS lines (Figures 1A–F). In comparison with those under NT, JA, and MeJA contents in stigmas were significantly increased at T1 (12:00 h on the first day during the temperature treatment), whereas they were significantly decreased at T2 (14:00 h on the first day during the temperature treatment), T3 (14:00 h on the second day during the temperature treatment), and T4 (14:00 h on the first day after the temperature treatment). The increase in JA and MeJA contents at T1 was more for Peiai 64S (increased by 19.6% for JA contents, by 31.5% for MeJA contents) than for Shen 08S (increased by 11.9% for JA contents, by 24.8% for MeJA contents) (Figures 1A–F). Decreases in JA and MeJA contents at T2, T3, and T4 were less for Peiai 64S (decreased by 18.3, 33.3, and 36.6% for JA contents, by 18.3, 29.2, and 46.8% for MeJA contents) for Shen 08S (decreased by 24.8, 38.1, and 41.4% for JA contents, by 42.6, 48.2, and 56.9% for MeJA contents) (Figures 1A–F).

The contents of H_2_O_2_ in stigmas were significantly increased at the four measurement times under HT when compared with those under NT for all the test rice lines (Supplementary Figures 1G–I). The increase in H_2_O_2_ contents was more for Shen 08S (increased by 69.6%, on average at T1 to T4 in the two study years) than for Peiai 64S (increased by 52.9%, on average at T1 to T4 in 2 years). In contrast, the catalase activity in stigmas was significantly increased at T1, but decreased at T2, T3, and T4 under HT, relative to that under NT (Supplementary Figures 1A–C). However, the catalase activity varied with rice lines. Peiai 64S (decreased by 20.3%, on average at T1 to T4 in the two study years) exhibited less decrease in catalase activity than Shen 08S (decreased by 34.6%, on average at T1 to T4 in the two study years) under HT (Supplementary Figures 1A–C). Similar to catalase activity, AsA content in stigmas was increased at T1 and decreased at T2, T3, and T4 under HT in comparison with that under NT (Supplementary Figures 1D–F). On average at T1 to T4 in both years, Peiai 64S exhibited higher AsA contents in stigmas (decreased by 22.9%) than Shen 08S (decreased by 26.7%), implying that Peiai 64S has higher antioxidant ability than Shen 08S.

### Exertion Rate, Fresh Weight, and the Area of Stigmas

Compared with NT, the HT significantly decreased exertion percentages of single, dual, and total stigmas of both PTSGMS rice lines (Table 1). Although Peiai 64S exhibited more decrease in the exertion percentage of single stigma (13.50–14.04%) than Shen 08S (10.11–10.12%), the former showed less decrease in exertion percentages of dual stigmas (5.49–6.63%) and total stigmas (8.26–9.22%) than the latter (13.92–14.40% for dual stigmas and 12.23–12.50% for total stigmas (Table 1). In contrast to those of the PTSGMS rice lines, the exertion percentages of single, dual, and total stigmas of the restorer line Yangdao 6 were all significantly increased under HT stress (Table 1).

**Table 1:** Effect of high-temperature stress during anthesis on stigma exertion of rice.

**Year**	**Line**	**Treatment**	**Exertion percentage of single stigma**	**Exertion percentage of dual stigmas**	**Exertion percentage of total stigmas**
2017	Peiai 64S	NT	31.4 ± 0.23e	59.0 ± 1.23a	90.4 ± 1.06a
		HT	27.2 ± 0.51f	55.8 ± 0.32b	83.0 ± 0.59c
	Shen 08S	NT	38.5 ± 1.26c	48.9 ± 1.67c	87.4 ± 2.93b
		HT	34.6 ± 0.60d	42.1 ± 0.42d	76.7 ± 0.80e
	Yangdao 6	NT	44.4 ± 0.81b	20.7 ± 0.72f	65.1 ± 0.13f
		HT	50.6 ± 0.04a	28.7 ± 1.32e	79.3 ± 1.29d
2018	Peiai 64S	NT	32.1 ± 0.54e	59.6 ± 0.90a	91.6 ± 0.88a
		HT	27.6 ± 0.51f	55.6 ± 1.28b	83.2 ± 1.56c
	Shen 08S	NT	39.0 ± 1.28c	49.3 ± 1.28c	88.3 ± 2.53b
		HT	35.1 ± 0.61d	42.2 ± 0.19d	77.3 ± 0.74e
	Yangdao 6	NT	45.1 ± 0.82b	21.0 ± 0.73f	66.1 ± 0.13f
		HT	51.4 ± 0.05a	28.4 ± 1.45e	79.8 ± 1.46d

*NT and HT represent normal temperature and high temperature, respectively. Data are expressed as the mean ± SD (n = 18). Different letters indicate significant difference at the P ≤ 0.05 level within the same column*.

**Figure 2:**
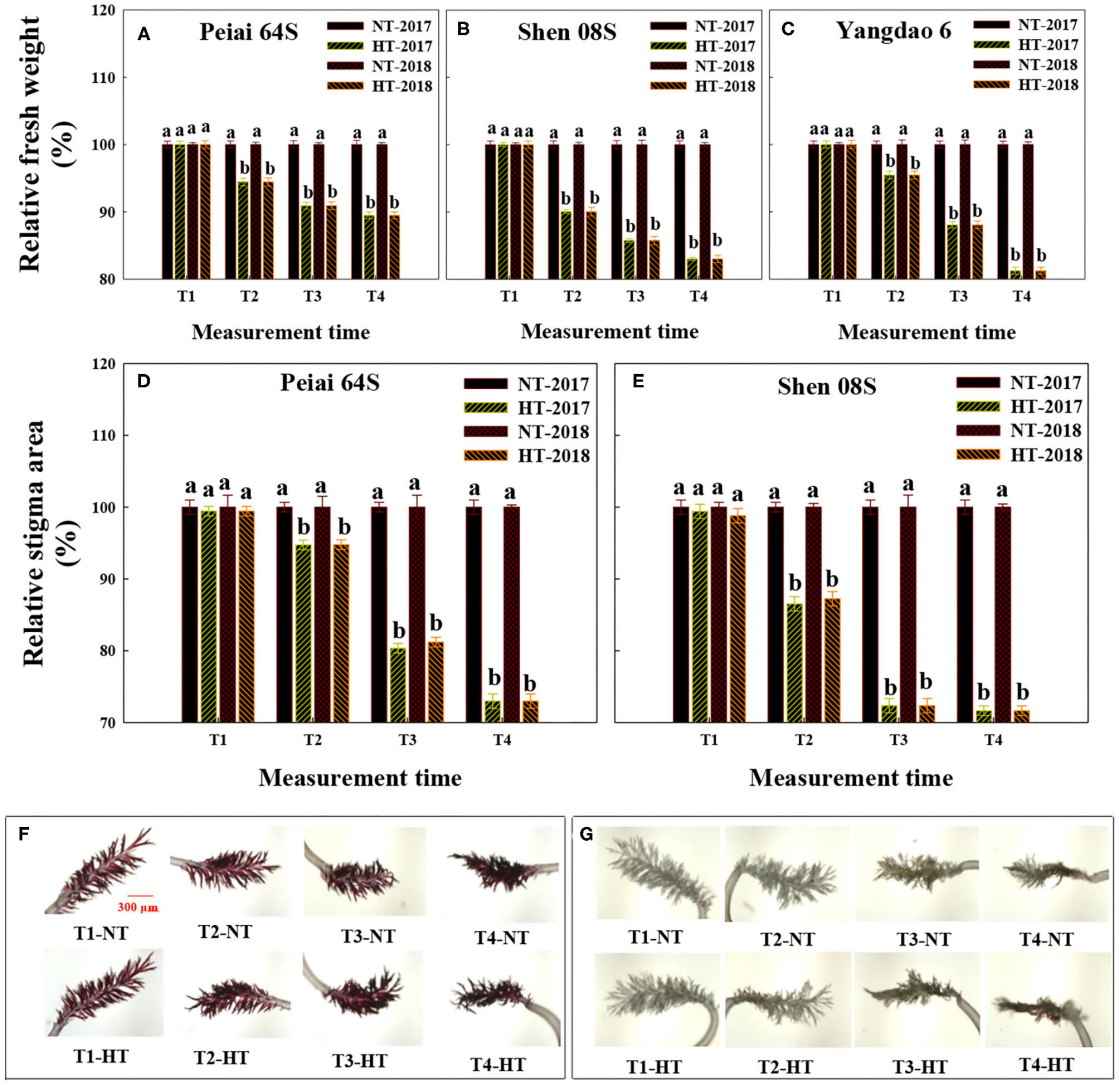
Effect of high-temperature stress during anthesis on the stigma fresh weight **(A–C)** and stigma area **(D–G)** of rice. NT and HT represent normal temperature and high temperature, respectively, and the NT is taken as control. Data are expressed as relative values, and those of controls are taken as 100%. Pictures in **(F,G)** are Peiai 64S and Shen 08S, respectively. T1: 12:00 h on the first day during the temperature treatment; T2: 14:00 h on the first day during the temperature treatment; T3: 14:00 h on the second day during the temperature treatment; T4: 14:00 h on the first day after the temperature treatment. The bars in **(A–E)** are SD of three biological replications. Different letters above the bars represent significant differences at the *P* ≤ 0.05 level within the same line and the same measurement time. The images of stigma area in **(F,G)** were taken at 50× magnifications.

The HT treatment significantly decreased stigma fresh weight of rice at T2, T3, and T4 in comparison with the NT (Figures 2A–C). The decrease in stigma fresh weight under HT varied with lines, with least for Peiai 64S (decreased by 8.39%) and the most for Shen 08S (decreased by 13.7%), on average (Figures 2A–C).

As shown in Figures 2D–G, the area of stigma of both PTSGMS lines exhibited no significant difference between NT and HT at T1. However, the stigma area was significantly decreased under HT at the measurement time of T2, T3, and T4 when compared with that under NT, with less decrease for Peiai 64S (decreased by 5.25% at T2, 19.2% at T3, and by 27.0% at T4) than that for Shen 08S (decreased by 13.1% at T2, 27.7% at T3, and by 28.4% at T4), on average in both years (Figures 2D–G, T2–4), indicating that Peiai 64S possesses stronger tolerance to HT stress during anthesis than Shen 08S.

### Pollen Germination on Stigmas and Seed Yield of Hybrids

Compared with NT, HT significantly decreased the number of pollens germinated on the stigma (the pollens on the stigmas of PTSGMS lines were from the restorer line) with variation among the 3 lines (Supplementary Figures 2A–G). On average, the reduction in pollen germination under HT was the most for Yangdao 6 (decreased by 53.36%), the least for Peiai 64S (decreased by 37.01%), and intermediate for Shen 08S (decreased by 47.37%) (Supplementary Figures 2A–G).

Table 2 shows the fertilization and seed-setting rates and seed yield of the hybrids produced by PTSGMS rice lines crossed with the restorer line Yangdao 6. As HT was conducted during anthesis, the spikelets per pot showed no significant difference between NT and HT for the same rice line. However, HT significantly reduced fertilization rate and, accordingly, significantly decreased seed-setting rate and seed yield in comparison with NT (Table 2). These reductions varied with hybrids or cultivars. For example, the seed yield under HT was reduced by 29.94 to 31.05% for Peiai 64S × Yangdao 6, by 53.09 to 54.32% for Shen 08S × Yangdao 6, and by 16.17 to 18.52% for Yangdao 6 when compared with that under respective NT. Compared with that under NT, 1,000-grain weight was significantly increased under HT (Table 2), indicating that the reduction in the seed yield under HT is mainly attributed to the reduction in the fertilization rate and the seed-setting rate.

**Table 2:** Effect of high-temperature stress during anthesis on the seed yield and its components of rice.

**Year/cultivar**	**Treatment**	**Spikelets per pot**	**Fertilization percentage (%)**	**Seed-setting percentage (%)**	**10^**3**^-grain weight (g)**	**Seed yield (g pot^**−1**^)**
**2017**						
Peiai 64S × Yangdao 6	NT	3,702 ± 142ab	56.5 ± 1.58e	54.8 ± 1.57c	20.4 ± 0.39d	39.9 ± 2.13d
	HT	3,741 ± 55.1a	37.5 ± 1.55f	36.1 ± 1.53e	21.4 ± 0.38c	27.5 ± 1.34f
Shen 08S × Yangdao 6	NT	3,227 ± 153d	47.9 ± 1.19c	46.4 ± 1.18d	18.7 ± 0.26f	26.6 ± 2.32c
	HT	3,185 ± 74.8d	22.5 ± 1.68d	21.3 ± 1.66f	19.7 ± 0.36e	12.2 ± 0.97e
Yangdao 6	NT	3,552 ± 158bc	95.4 ± 0.77a	93.3 ± 0.76a	29.8 ± 0.35b	96.5 ± 3.93a
	HT	35,49 ± 81.7c	76.0 ± 2.24b	74.1 ± 2.22b	30.9 ± 0.07a	78.6 ± 2.75b
**2018**						
Peiai 64S × Yangdao 6	NT	3,774 ± 89.3a	55.8 ± 1.11e	54.2 ± 1.10c	20.6 ± 0.26d	40.7 ± 0.71d
	HT	3,814 ± 63.1a	37.8 ± 1.42f	36.4 ± 1.40e	21.5 ± 0.40c	28.5 ± 1.20f
Shen 08S × Yangdao 6	NT	3,260 ± 108d	47.8 ± 1.19c	46.3 ± 1.18d	18.6 ± 0.33f	26.7 ± 1.49c
	HT	3,194 ± 75.2d	23.0 ± 0.94	21.7 ± 0.93f	19.8 ± 0.36e	12.5 ± 0.79e
Yangdao 6	NT	3,557 ± 89.2bc	95.6 ± 0.76a	93.5 ± 0.75a	29.5 ± 0.35b	96.0 ± 1.69a
	HT	3,589 ± 164bc	76.6 ± 2.20b	74.7 ± 2.18b	30.8 ± 0.39a	80.5 ± 3.78b

*NT and HT represent normal temperature and high temperature, respectively. Data are expressed as the mean ± SD (n = 5). Different letters indicate significant difference at the P ≤ 0.05 level within the same column*.

Correlation analysis showed that the contents of both JA and MeJA in stigmas were very significantly and positively correlated with the fertilization rate and the seed-setting rate with *r* = 0.976^**^ to 0.992^**^ (Table 3). Contents of JA and MeJA were also significantly and positively correlated with the morphological and physiological traits that reflect stigma vitality, such as the exertion rate of stigmas, stigma fresh weight, and catalase activity and AsA content in the stigma (*r* = 0.848^*^ to 0.980^**^), whereas they were negatively correlated with H_2_O_2_ content in stigmas with *r* = −0.853^*^ to −0.890^*^ (Table 3), implying that JAs may mediate the effect of HT during anthesis on stigma vitality of PTSGMS lines and consequently regulate fertilization rate and the seed-setting rate when the PTSGMS lines are crossed with a restorer line.

**Table 3:** Correlations of the jasmonic acid (JA) and methyl jasmonate (MeJA) contents with stigma vitality traits of rice.

**Correlation with**	**JA**	**MeJA**
Exertion percentage of single stigma	−0.140	−0.075
Exertion percentage of dual stigmas	0.879*	0.848*
Exertion percentage of total stigma	0.973**	0.987**
Stigma fresh weight	0.935**	0.905**
H_2_O_2_ contents in the stigma	−0.853*	−0.890*
CAT activity in the stigma	0.983**	0.964**
AsA contents in the stigma	0.980**	0.985**
Number of pollens germinated on single stigma	0.959**	0.937**
Fertilization percentage	0.981**	0.992**
Seed-setting percentage	0.976**	0.984**

Data for correlation analysis are from Tables 1, 2, Figures 1, 2, and Supplementary Figures 1, 2. *Correlation significance at the P ≤ 0.05 level (n = 6). **Correlation significance at the P ≤ 0.01 level (n = 6).

### Effect of MeJA Application

**Figure 3:**
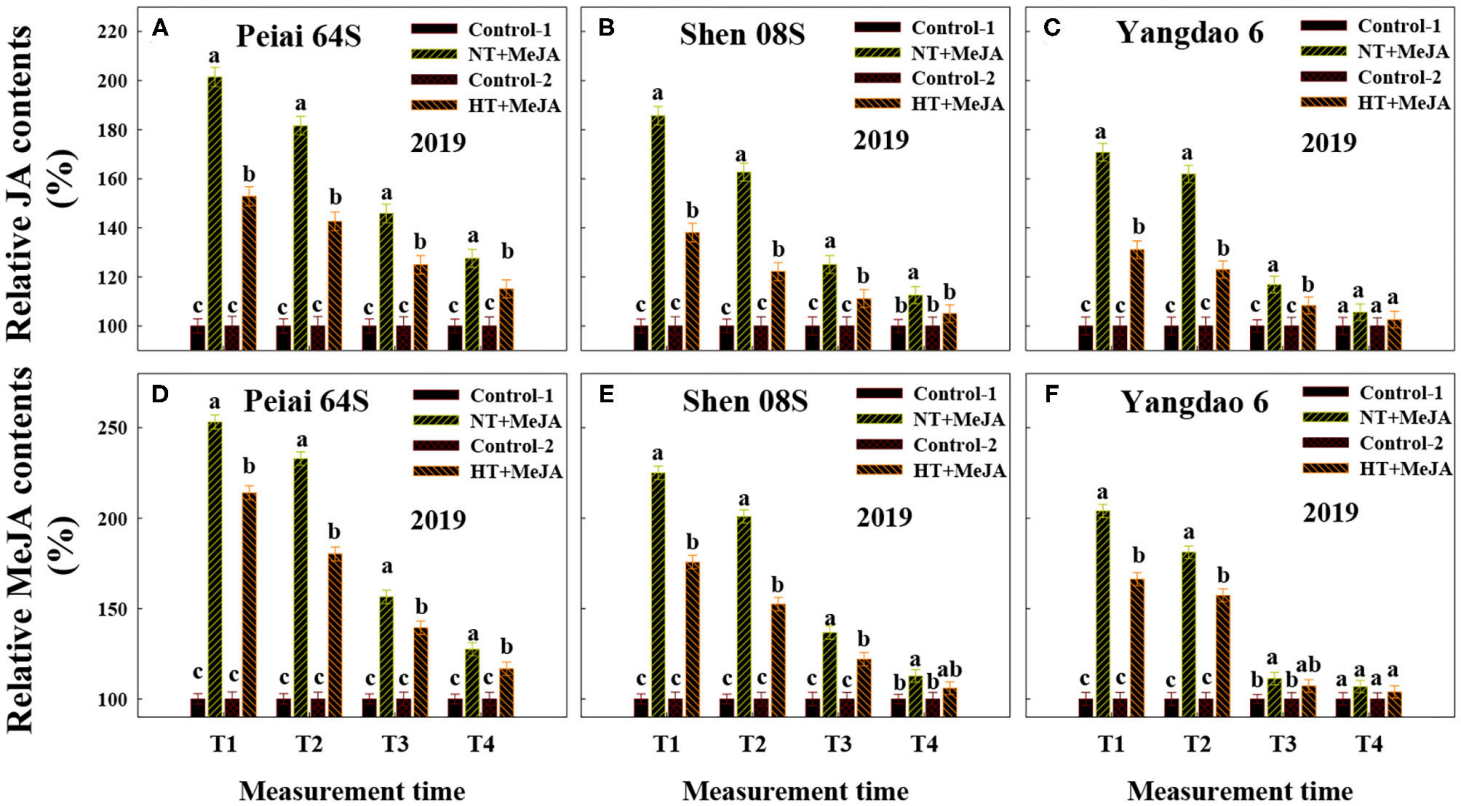
Effect of MeJA application on the contents of JA **(A–C)** and MeJA **(D–F)** in stigmas of rice subjected to high-temperature stress during anthesis. Control-1 and Control-2 indicate spraying water under normal temperature (NT) and spraying water under high temperature (HT), respectively. NT+MeJA and HT+MeJA represent spraying MeJA under NT and spraying MeJA under HT, respectively. Data are expressed as relative values, and those of controls are taken as 100%. T1: 12:00 h on the first day during the temperature treatment; T2: 14:00 h on the first day during the temperature treatment; T3: 14:00 h on the second day during the temperature treatment; T4: 14:00 h on the first day after the temperature treatment. The bars are SD of three biological replications. Different letters above the bars represent significant differences at the *P* ≤ 0.05 level within the same line and the same measurement time.

Compared with the Control-1 (spraying water under NT) and Control-2 (spraying water under HT), spraying MeJA under NT (NT+MeJA) and spraying MeJA under HT (HT+MeJA) significantly increased JA and MeJA contents in stigmas at 12:00 h (T1) and 14:00 h (T2) on the first day during the temperature treatment and 14:00 h on the second day during the temperature treatment (T3) for all the test rice lines (Figures 3A–F). Contents of JA and MeJA in stigmas of the two PTSGMS rice lines also showed some increase at 14:00 h on the first day after the temperature treatment (T4) when MeJA was applied, although their differences were not always significant between MeJA application and the controls (Figures 3A–F).

**Figure 4:**
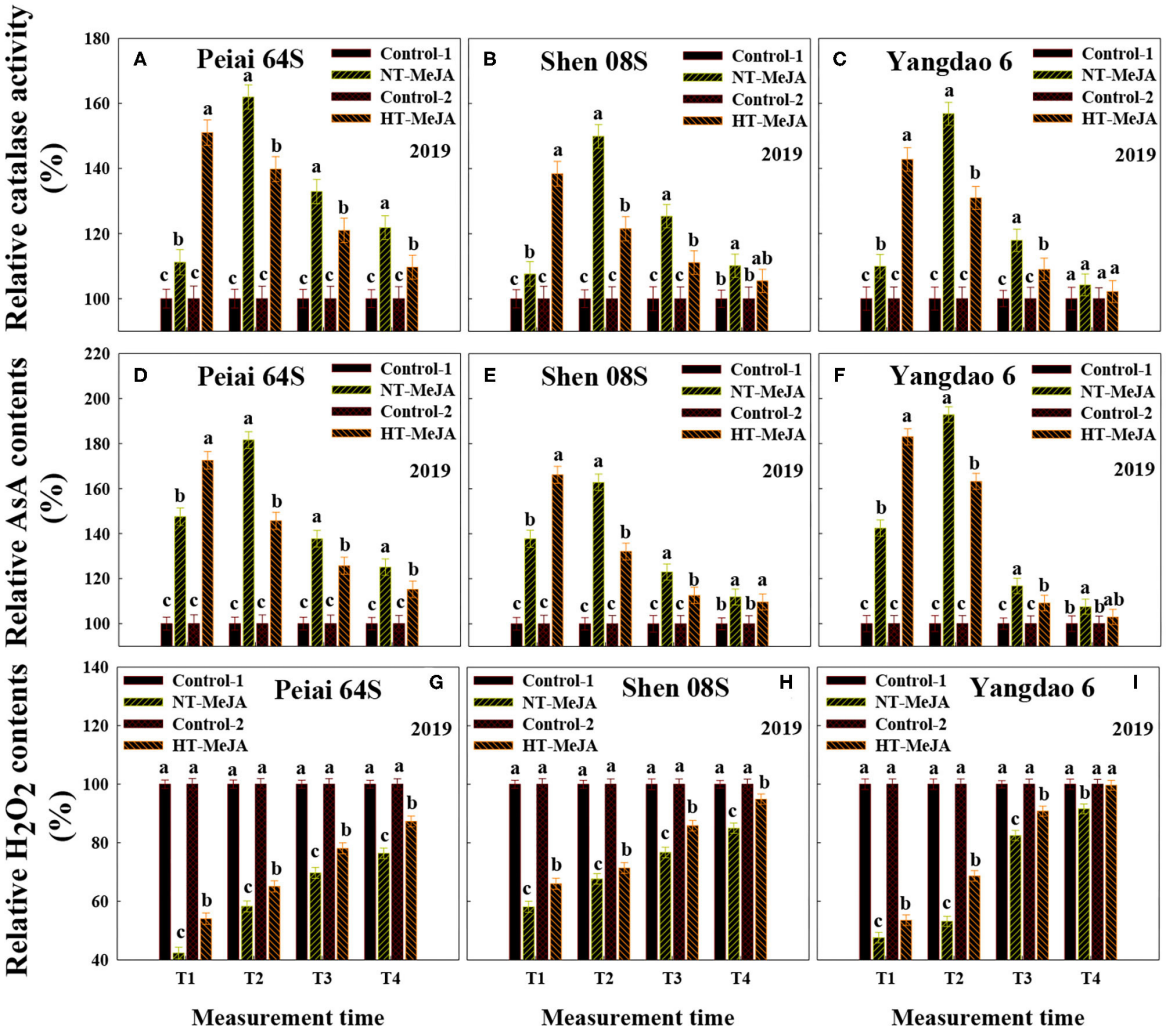
Effect of MeJA application on the catalase activity **(A–C)** and the contents of ascorbic acid (AsA) **(D–F)** and H_2_O_2_ **(G–I)** in stigmas of rice subjected to high-temperature stress during anthesis. Control-1 and Control-2 indicate spraying water under normal temperature (NT) and spraying water under high temperature (HT), respectively. NT+MeJA and HT+MeJA represent spraying MeJA under NT and spraying MeJA under HT, respectively. Data are expressed as relative values, and those of controls are taken as 100%. T1: 12:00 h on the first day during the temperature treatment; T2: 14:00 h on the first day during the temperature treatment; T3: 14:00 h on the second day during the temperature treatment; T4: 14:00 h on the first day after the temperature treatment. The bars are SD of three biological replications. Different letters above the bars represent significant differences at the *P* ≤ 0.05 level within the same line and the same measurement time.

Similar to JA and MeJA contents, catalase activity and AsA contents in stigmas were significantly increased for both PTSGMS lines under NT+MeJA and HT+MeJA, especially under HT+MeJA when compared with those under Conrol-1 and Control-2, (Figures 4A,B,D,E). Applying MeJA also increased both catalase activity and AsA contents in stigmas of the restorer line Yangdao 6, but the increase was not always significant in comparison with the controls (Figures 4C,F).

**Figure 5:**
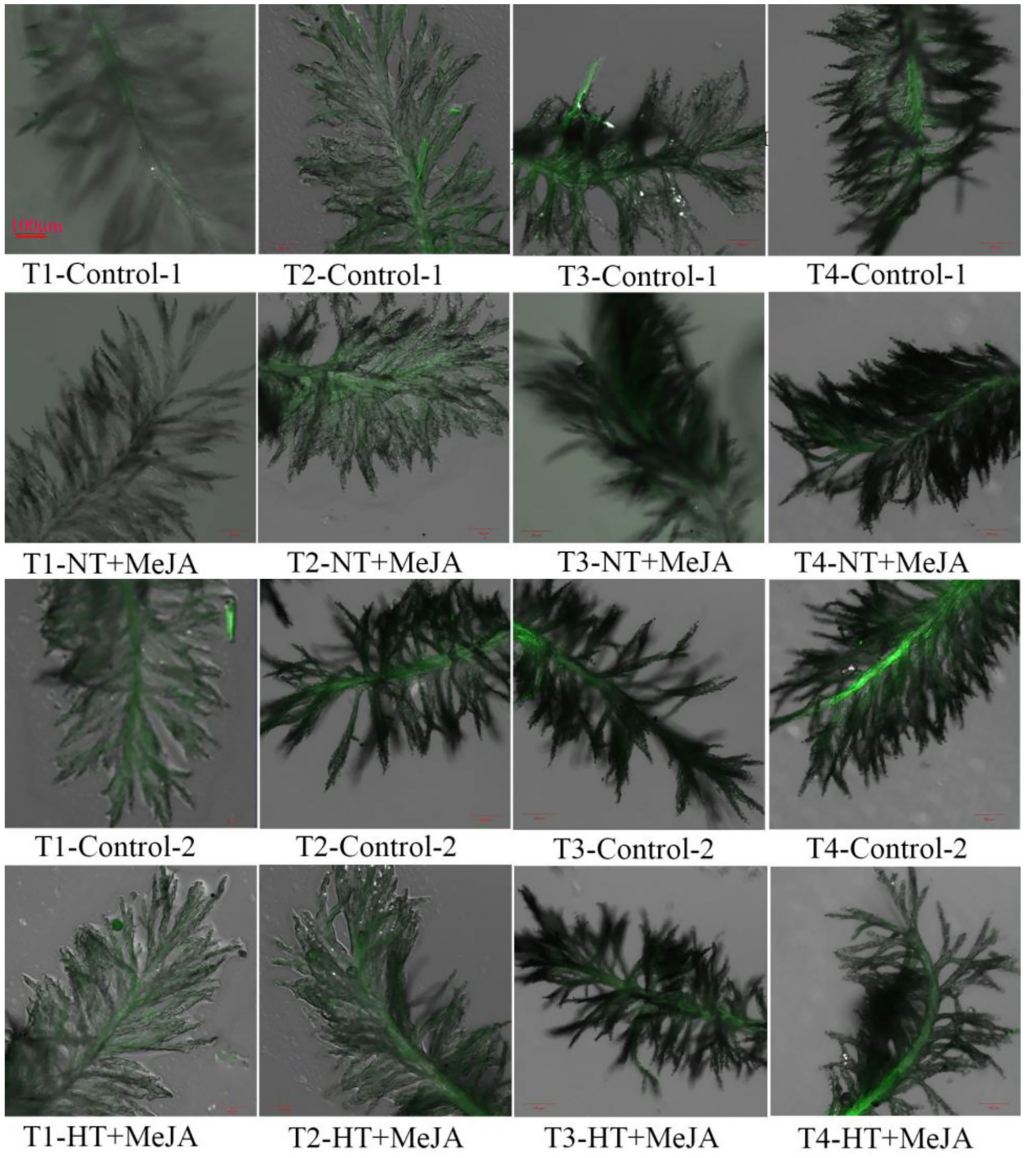
Effect of MeJA application on the accumulation of reactive oxygen species (ROS) in stigmas of Shen 08S subjected to high-temperature stress during anthesis. Control-1 and Control-2 indicate spraying water under normal temperature (NT) and spraying water under high temperature (HT), respectively. NT+MeJA and HT+MeJA represent spraying MeJA under NT and spraying MeJA under HT, respectively. T1: 12:00 h on the first day during the temperature treatment; T2: 14:00 h on the first day during the temperature treatment; T3: 14:00 h on the second day during the temperature treatment; T4: 14:00 h on the first day after the temperature treatment. Black unstained stigmas indicate an absence of ROS, and green-stained stigmas indicate the presence of ROS. The intensity of the green color is proportional to the ROS level. The stigmas were stained with the LIVE Green ROS detection kit and were taken at 60× magnifications.

**Figure 6:**
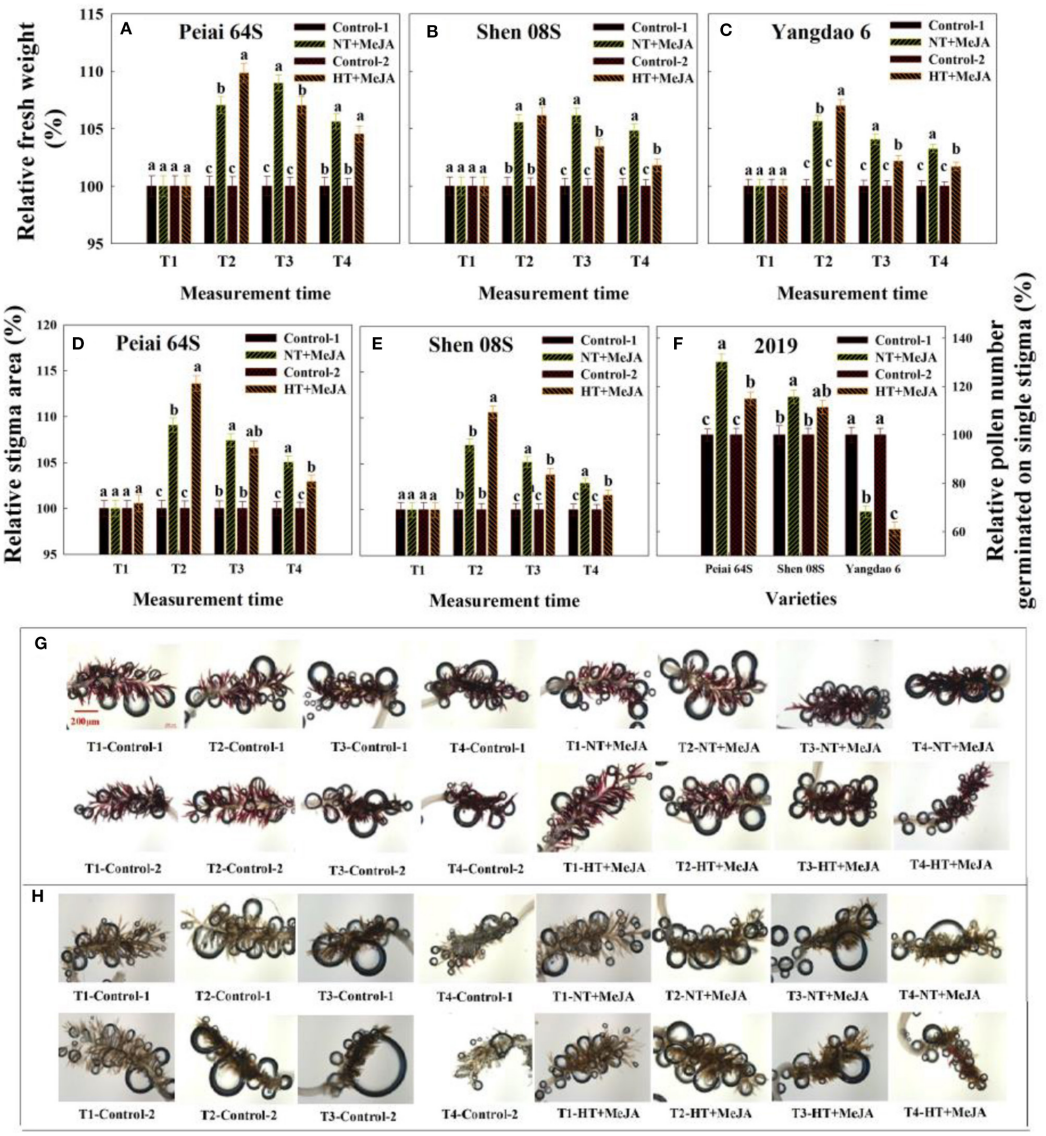
Effect of MeJA application on the stigma fresh weight **(A–C)**, stigma area **(D,E)**, pollen number germinated on the stigma **(F)**, and stigma receptivity **(G,H)** subjected to high-temperature stress during anthesis. Control-1 and Control-2 indicate spraying water under normal temperature (NT) and spraying water under high temperature (HT), respectively. NT+MeJA and HT+MeJA represent spraying MeJA under NT and spraying MeJA under HT, respectively. T1: 12:00 h on the first day during the temperature treatment; T2: 14:00 h on the first day during the temperature treatment; T3: 14:00 h on the second day during the temperature treatment; T4: 14:00 h on the first day after the temperature treatment. The bars in **(A–F)** are SD of three biological replications. Different letters above the bars represent significant differences at the *P* ≤ 0.05 level within the same line and the same measurement time. The release of air bubbles in **(G,H)** indicates that the stigma is receptive. The more the air bubbles are, the higher the stigma receptive is. The stigmas were stained with 3% (vol/vol) H_2_O_2_ solution and were taken at 50× magnifications.

The content of H_2_O_2_ in stigmas was decreased by applying MeJA, and the decrease was significant under HT, relative to the control (Figures 4G–I). Consistent with the decrease in H_2_O_2_ contents, ROS accumulation in stigmas was markedly reduced by MeJA application vs. the control (Figure 5). Opposite to ROS accumulation, the stigma receptivity reflecting stigma vitality was enhanced by the MeJA application in comparison with the control (Figures 6G,H).

Relative to control, application of MeJA increased sigma fresh weight and stigma area of Peiai 64S and Shen 08S at T2 to T4 under HT (Figures 6A–E). Either stigma fresh weight or stigma area showed no significant difference between MeJA application and the controls at T1 (Figures 6A–E).

The effect of MeJA application on the pollen germination on stigmas varied with rice lines (Figure 6F). In comparison with that under control, the pollen number germinated on single stigma under NT+MeJA and HT+MeJA was significantly increased for Peiai 64S, but no significant difference was observed for Shen 08S between Control-2 and HT+MeJA. Unexpectedly, the pollen number was significantly decreased for Yangdao 6 under either NT+MeJA or HT+MeJA in comparison with the Control-1 or Control-2 (Figure 6F).

Compared with Control-1 and Control-2, both NT+MeJA and HT+MeJA treatments increased exertion percentages of single stigma, dual, and total stigmas for all the test lines either significantly or insignificantly (Table 4). Applying MeJA significantly increased seed yield by 8.7 to 11.1% under NT and by 30.7 to 54.4% under HT for both hybrids of Peiai 64S × Yangdao 6 and Shen 08S × Yangdao 6 when compared with the respective control (Table 5). The increase in the seed yield for both hybrids was attributed to the increases in fertilization percentage and seed-setting percentage. On the other hand, application of MeJA significantly decreased the seed yield of Yangdao 6 due to the reduction of fertilization percentage and seed-setting percentage (Table 5).

**Table 4:** Effect of MeJA application on the stigma exertion of rice subjected to high-temperature stress during anthesis in 2019.

**Line**	**Treatment**	**Exertion percentage of single stigma**	**Exertion percentage of dual stigmas**	**Exertion percentage of total stigmas**
Peiai 64S	Control-1	31.7 ± 0.42h	60.3 ± 1.26b	92.0 ± 1.67b
	NT+MeJA	32.1 ± 0.81h	64.0 ± 0.73a	96.1 ± 1.30a
	Control-2	27.7 ± 0.52i	57.0 ± 0.32c	84.7 ± 0.61d
	HT+MeJA	31.0 ± 0.47h	61.3 ± 1.02b	92.3 ± 1.38b
Shen 08S	Control-1	39.9 ± 0.50e	50.0 ± 1.70d	89.9 ± 1.61c
	NT+MeJA	40.5 ± 1.30e	51.2 ± 0.80d	91.8 ± 2.02b
	Control-2	35.3 ± 0.62g	43.0 ± 0.43f	78.3 ± 0.82f
	HT+MeJA	37.0 ± 0.28f	47.5 ± 0.59e	84.6 ± 0.31d
Yangdao 6	Control-1	45.4 ± 0.83d	21.2 ± 0.73j	66.5 ± 0.13h
	NT+MeJA	47.8 ± 1.51c	23.7 ± 0.77i	71.5 ± 0.96g
	Control-2	51.7 ± 0.05b	29.3 ± 1.35h	80.9 ± 1.32e
	HT+MeJA	54.9 ± 0.85a	34.6 ± 1.36g	89.5 ± 1.96c

*Control-1 and Control-2 indicate spraying water under normal temperature (NT) and spraying water under high temperature (HT), respectively. NT+MeJA and HT+MeJA represent spraying MeJA under NT and spraying MeJA under HT, respectively. Data are expressed as the mean ± SD (n = 18). Different letters indicate significant difference at the P ≤ 0.05 level within the same column*.

**Table 5:** Effect of MeJA application on the seed yield and its components of rice subjected to high-temperature stress during anthesis in 2019.

**Year/Cultivar**	**Treatment**	**Spikelets per pot**	**Fertilization percentage (%)**	**Seed-setting percentage (%)**	**10^**3**^-grain weight (g)**	**Seed yield (g pot^**−1**^)**
Peiai 64S × Yangdao 6	Control-1	3,743 ± 91.4a	55.3 ± 1.91f	54.0 ± 1.95f	19.9 ± 0.16f	38.7 ± 0.73f
	NT+MeJA	3,740 ± 61.6a	66.0 ± 0.93e	62.9 ± 1.00e	18.9 ± 0.43g	43.0 ± 2.02e
	Control-2	3,721 ± 54.5a	38.6 ± 0.77j	36.9 ± 0.76j	21.4 ± 0.38d	27.7 ± 1.32h
	HT+MeJA	3,761 ± 63.2a	52.0 ± 0.90g	49.0 ± 1.47g	20.7 ± 0.40e	36.2 ± 0.65g
Shen 08S × Yangdao 6	Control-1	3,121 ± 33.0c	48.0 ± 1.19h	43.4 ± 1.47h	18.7 ± 0.26g	25.4 ± 0.82i
	NT+MeJA	3,094 ± 160.8c	54.5 ± 1.11f	52.8 ± 1.00f	18.0 ± 0.43h	27.6 ± 1.84h
	Control-2	3,059 ± 113.8c	22.0 ± 1.83k	20.7 ± 1.80k	19.7 ± 0.36f	11.4 ± 1.13k
	HT+MeJA	3,105 ± 89.1c	34.0 ± 1.07i	32.4 ± 1.06i	18.6 ± 0.40g	17.6 ± 0.74j
Yangdao 6	Control-1	3,470 ± 61.0b	95.4 ± 0.77a	93.3 ± 0.76a	30.2 ± 0.30bc	96.7 ± 1.98a
	NT+MeJA	3,500 ± 82.9b	89. ± 1.52b	87.3 ± 1.00b	30.1 ± 0.43c	91.0 ± 2.01b
	Control-2	3,467 ± 48.2b	78.4 ± 0.62c	76.5 ± 0.61c	30.6 ± 0.36ab	79.9 ± 2.15c
	HT+MeJA	3,452 ± 62.4b	71.4 ± 1.15d	69.6 ± 1.47d	30.9 ± 0.40a	73.1 ± 1.72d

*Control-1 and Control-2 indicate spraying water under normal temperature (NT) and spraying water under high temperature (HT), respectively. NT+MeJA and HT+MeJA represent spraying MeJA under NT and spraying MeJA under HT, respectively. Data are expressed as the mean ± SD (n = 5). Different letters indicate significant difference at the P ≤ 0.05 level within the same column*.

## Discussion

Although JAs have been proposed to play important roles in responses to biotic and abiotic stresses (Cheng et al., 2009; Kazan, 2015; Asghari, 2019; Nguyen et al., 2019; Ali and Baek, 2020; Chen et al., 2020; Yang et al., 2020), the information is unavailable prior to this study to show whether and how JAs in stigmas of PTSGMS rice lines could respond to HT stress during anthesis and thereby regulate stigma vitality. The results herein showed that a PTSGMS line with higher contents of JA and MeJA in the stigmas under HT stress had better traits reflecting stigma vitality, such as heavier fresh weight of stigmas, larger stigma area, greater exertion rate of stigmas and more pollen number geminated on the stigma (Figures 1, 2; Supplementary Figure 2; Table 1), leading to higher fertilization percentage, seed-setting percentage, and seed yield when the line was crossed with a restorer rice line (Table 2). Contents of both JA and MeJA were observed to be correlated significantly and positively with these traits (Table 3). Furthermore, increases in JA and MeJA contents in stigmas by applying MeJA during anthesis significantly improved the traits related to stigma vitality (Figures 3, 6; Tables 4, 5). Therefore, we speculate that JAs (JA and MeJA) could alleviate the adverse effect of HT stress on stigma vitality of PTSGMS rice lines and, accordingly, reduce the losses of their hybrid seed yields subjected to HT stress during anthesis.

The mechanism by which JAs assuage the harm of HT stress to the stigmas of PTSGMS rice lines is not fully understood. Generally, HT stress could increase ROS and reduce antioxidants in plant organs, and excessive production and accumulation of ROS could damage cell membranes and cause programmed cell death (Aebi, 1984; Hoang et al., 2019). It is suggested that H_2_O_2_ is the most stable and the least reactive ROS among the ROS, and it can easily cross the membrane to damage cells (Yang and Poovaiah, 2002; Quan et al., 2008). Catalase is considered as the first antioxidant enzyme to scavenge H_2_O_2_ generated during different pathways under normal and stress conditions and plays a vital role in maintaining redox balance in plant cells (Loew, 1900; Corpas et al., 2001; Mittler, 2002; Foyer and Noctor, 2005). On other hand, AsA is considered as the most versatile among all antioxidant molecules as it can scavenge all types of ROS and regenerate other antioxidants (Li and Van Staden, 1998; Ai et al., 2008). It is reported that JAs could activate the defense system of plants, mainly via enhancing activities of antioxidative enzymes, such as catalase and glutathione peroxidase, and increasing other defensive compounds such as AsA and heat shock proteins, thereby suppressing ROS generation and reducing programmed cell death under stresses (Hasanuzzaman et al., 2013; Farhangi-Abriz and Ghassemi-Golezani, 2019; Chen et al., 2020; Yang et al., 2020). The present results showed that a PTSGMS line with higher contents of JA and MeJA in the stigmas under HT stress exhibited lower H_2_O_2_ contents and higher AsA contents and catalase activity there (Figure 1 and Supplementary Figure 1). Contents of JA and MeJA were negatively correlated with H_2_O_2_ contents, whereas they were positively correlated with AsA contents and catalase activity (Table 3). Applying MeJA to panicles under HT stress significantly decreased H_2_O_2_ contents and ROS accumulation, whereas it significantly increased AsA contents and catalase activity in stigmas and increased stigma receptivity (Figures 3–5, 6G,H). These results demonstrate that JAs play vital roles in strengthening AOS and suppressing ROS production in rice stigmas and thereby alleviating the injury to stigmas caused by HT stress during anthesis.

The main findings of the present study are as follows: (1) JAs contents in stigmas varied with PTSGMS rice lines, and a PTSGMS rice line with higher contents of JAs in stigmas exhibited stronger thermotolerance; (2) applying MeJA could alleviate stigma impairment caused by HT stress during anthesis of PTSGMS lines via maintaining the redox homeostasis. These findings would have great significance in the production of “two-line method” hybrid rice. First, a rice cultivar or a PTSGMS rice line with strong thermotolerance could be bred by enhancing JAs biosynthesis through classical breeding methods and/or genetic engineering technology. Second, MeJA could be used as a chemical regulator to alleviate HT stress on the stigmas of PTSGMS rice lines by inhibiting ROS and enhancing AOS. Moreover, there is a report showing that a cytoplasmic male sterile rice line treated with MeJA under NT during anthesis substantially increased the percentage of filled spikelets and the seed yield when the line was crossed with a restorer line (Zeng et al., 1999; Liu et al., 2017). The results suggest that application of MeJA is a practice not only to cope with HT stress, but also to increase seed yield in the hybrid rice production under the NT condition.

It should be noteworthy that the stigma exertion of the restorer line of Yangdao 6 under HT stress is different from that of the PTSGMS line of Peiai 64S or Shen 08S (Tables 1, 4). The probable explanation is that the two types of rice lines have genetic difference in pollination habits. Yangdao 6 is a totally male fertile rice line, whose floral structure is originally beneficial to self-pollination when the stigmas are not exerted. HT stress during anthesis could decrease self-pollination and promote more stigma exertion for pollination (Yuan, 1997; Ma and Yuan, 2015; Mou, 2016), even though HT stress decreased the stigma physiological activity, which was evidenced by the decreases in contents of JAs and AsA and the activity of catalase and increases in H_2_O_2_ contents in stigmas (Figure 1, Supplementary Figure 1). On the other hand, the PTSGMS rice lines can only finish the fertilization by the way of cross-pollination with the male fertile rice line, and the stigma exertion is necessary for cross-pollination. HT stress during anthesis decreases the stigma exertion and, accordingly, decreases pollination (Yuan, 1997; Ma and Yuan, 2015; Mou, 2016). Further research is needed to understand the mechanism underlying the difference in the stigma exertion between the PTSGMS line and the restorer line under HT stress during anthesis.

It is also notable that applying MeJA to the restorer line Yangdao 6 (an indica inbred) showing normal male fertility significantly decreased fertilization rate and seed-setting rate under both NT and HT during anthesis (Table 5). A similar observation is also reported on a japonica inbred by Kobayasi and Atsuta (2010). The mechanism underlying the decreases in fertilization rate and seed-setting rate by applying MeJA remains unclear. A probable explanation is that application of MeJA to a male fertile inbred rice cultivar during anthesis could result in pollen sterility due to MeJA-induced early opening of flowers that are expected to open on the next day (Kobayasi and Atsuta, 2010). As a PTSGMS rice line exhibits totally male sterility when temperature is greater than 23°C during anthesis (Yuan, 1997; Ma and Yuan, 2015; Mou, 2016), application of MeJA to such a line could not affect pollen fertility. Therefore, we argue that applying MeJA during anthesis may benefit PTSGMS rice lines by enhancing stigma vitality, whereas it produces an adverse effect on a male fertile inbred rice cultivar/line by reducing pollen fertility. It merits to investigate the mechanism by which application of MeJA during anthesis reduces pollen fertility of a male fertile inbred.

It is suggested that MeJA could trigger cleistogamy sometimes in cereals, which can reduce sterility under heat stress conditions (Honda et al., 2006; Koike et al., 2015). As mentioned previously, a PTSGMS rice line shows totally male sterility under HT during anthesis; cleistogamy could not occur as MeJA is applied to PTSGMS rice lines under HT stress. It is also worthy of mention that JA level has been observed to be decreased in rice seedling leaves under heat stress (Du et al., 2013). The present results showed that both JA and MeJA contents in rice stigmas were increased at the first measurement time and decreased at other measurement times (Figure 3). The results imply that the decrease or increase in JAs levels in plants may depend on plant organs and growth stages, the treatment time, and the duration of HT stress. Further research is needed to verify the roles of JAs in responding to HT stress under different conditions including genetic backgrounds, plant growth stages, plant tissues, and environmental factors.

## Conclusion

A PTSGMS line with higher JA and MeJA contents in stigmas under HT stress had better traits reflecting stigma vitality including higher AsA content and catalase activity and lower H_2_O_2_ content in the stigmas, heavier fresh weight of stigmas, larger single stigma area, greater exertion rate of stigmas, and more number of pollens geminated on the stigma, leading to higher seed yield when the line was crossed with a restorer rice line. JAs could alleviate the injury to stigmas caused by HT stress during anthesis through strengthening AOS and suppressing ROS production in the stigmas of PTSGMS rice lines. A higher level of JAs in the stigmas under HT stress could be used as a criterion or a physiological trait for breeding and selecting a PTSGMS line with thermotolerance, and MeJA could be used as a chemical regulator to minimize the adverse effect of HT stress on the stigma vitality of PTSGMS rice lines. Further work is needed to verify the roles of JAs in responding to HT stress under different conditions including different genetic backgrounds and to understand the molecular mechanism by which JAs assuage the harm of HT stress to stigma vitality of rice.

## Data Availability Statement

The raw data supporting the conclusions of this article will be made available by the authors, without undue reservation.

## Author Contributions

JC and JY designed the research. JC, WM, KF, HS, YZ, YS, CL, and JH performed the research. JC, KF, KZ, and ZW analyzed the data. JC and JY wrote the paper. All authors reviewed the manuscript.

## Conflict of Interest

The authors declare that the research was conducted in the absence of any commercial or financial relationships that could be construed as a potential conflict of interest.

## Supplementary Material

Supplementary Figure 1 Effect of high-temperature stress during anthesis on the catalase activity **(A–C)**, the contents of ascorbic acid (AsA) **(D–F)**, and H_2_O_2_ **(G–I)** in stigmas of rice. NT and HT represent normal temperature and high temperature, respectively, and the NT is taken as control. Data are expressed as relative values, and those of controls are taken as 100%. T1: 12:00 h on the first day during the temperature treatment; T2: 14:00 h on the first day during the temperature treatment; T3: 14:00 h on the second day during the temperature treatment; T4: 14:00 h on the first day after the temperature treatment. The bars are SD of three biological replications. Different letters above the bars represent significant differences at the *P* ≤ 0.05 level within the same line and the same measurement time.

Supplementary Figure 2 Effect of high-temperature stress during anthesis on the number of pollens germinated on stigma of rice. NT and HT represent normal temperature and high temperature, respectively. Luminous circles in **(A–F)** indicate germinated pollens. Data in G are expressed as relative values, and those of the control (NT) are taken as 100%. The bars in G are SD of six biological replications. Different letters above the bars represent significant differences at the *P* ≤ 0.05 level within the same line. The images of pollens germinated on single stigma were taken at 50× magnifications.

## Abbreviations

AsA: ascorbic acid
AOS: antioxidant system
HT: high temperature
JA: jasmonic acid
JAs: jasmonates
MeJA: methyl jasmonate
NT: normal temperature
PTSGMS: photothermosensitive genic male sterile
ROS: reactive oxygen species.
