# Supplementary figures and images for: Jasmonates Alleviate the Harm of High-Temperature Stress During Anthesis to Stigma Vitality of Photothermosensitive Genetic Male Sterile Rice Lines
Source: Front Plant Sci. 2021 Mar 29;12:634959. doi: 10.3389/fpls.2021.634959 (PMC8039518; doi:10.3389/fpls.2021.634959)

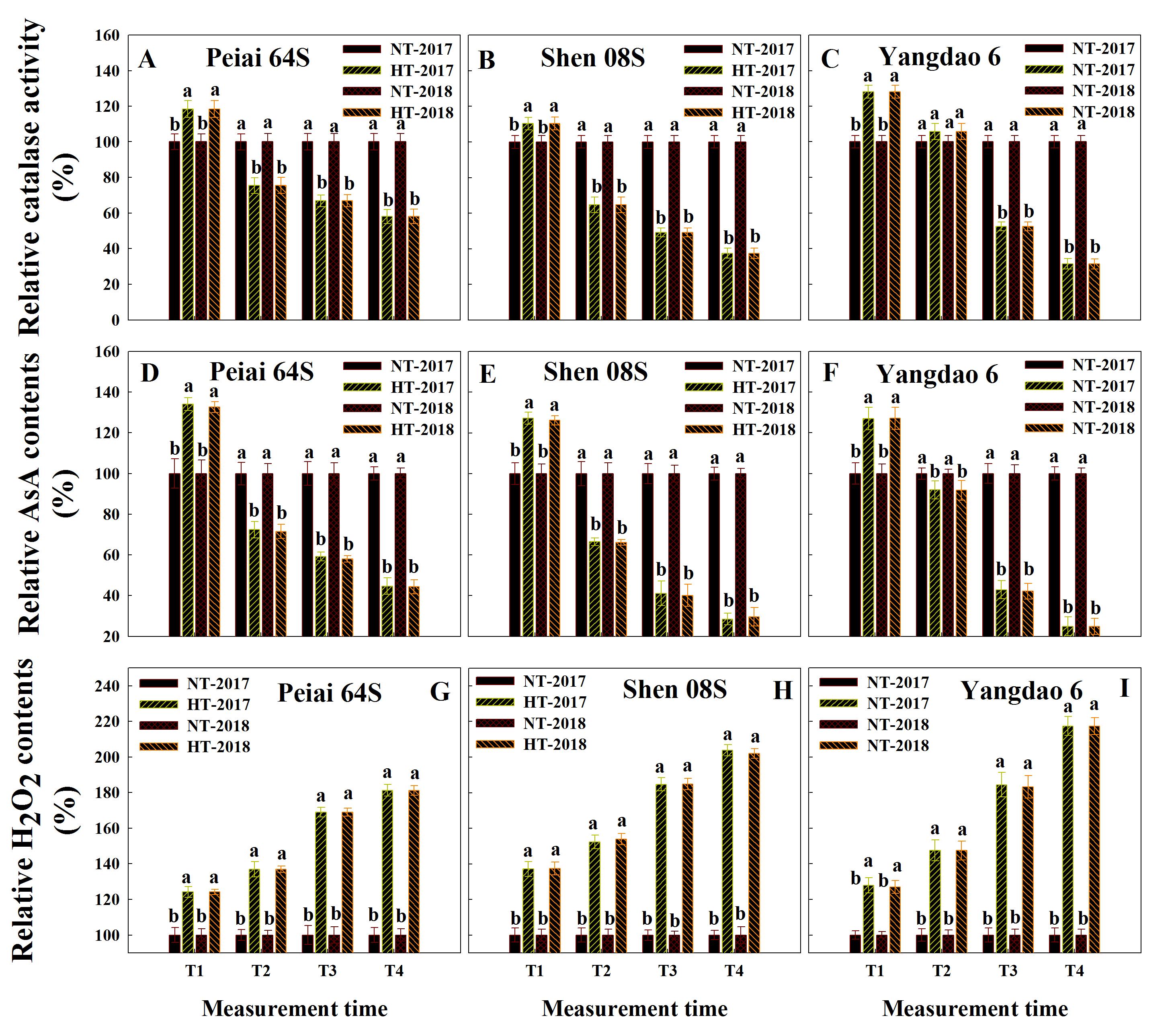

Supplement: Supplementary Figure 1 — Effect of high-temperature stress during anthesis on the catalase activity (A–C), the contents of ascorbic acid (AsA) (D–F), and H2O2 (G–I) in stigmas of rice. NT and HT represent normal temperature and high temperature, respectively, and the NT is taken as control. Data are expressed as relative values, and those of controls are taken as 100%. T1: 12:00 h on the first day during the temperature treatment; T2: 14:00 h on the first day during the temperature treatment; T3: 14:00 h on the second day during the temperature treatment; T4: 14:00 h on the first day after the temperature treatment. The bars are SD of three biological replications. Different letters above the bars represent significant differences at the P ≤ 0.05 level within the same line and the same measurement time. [file Image_1.jpg]

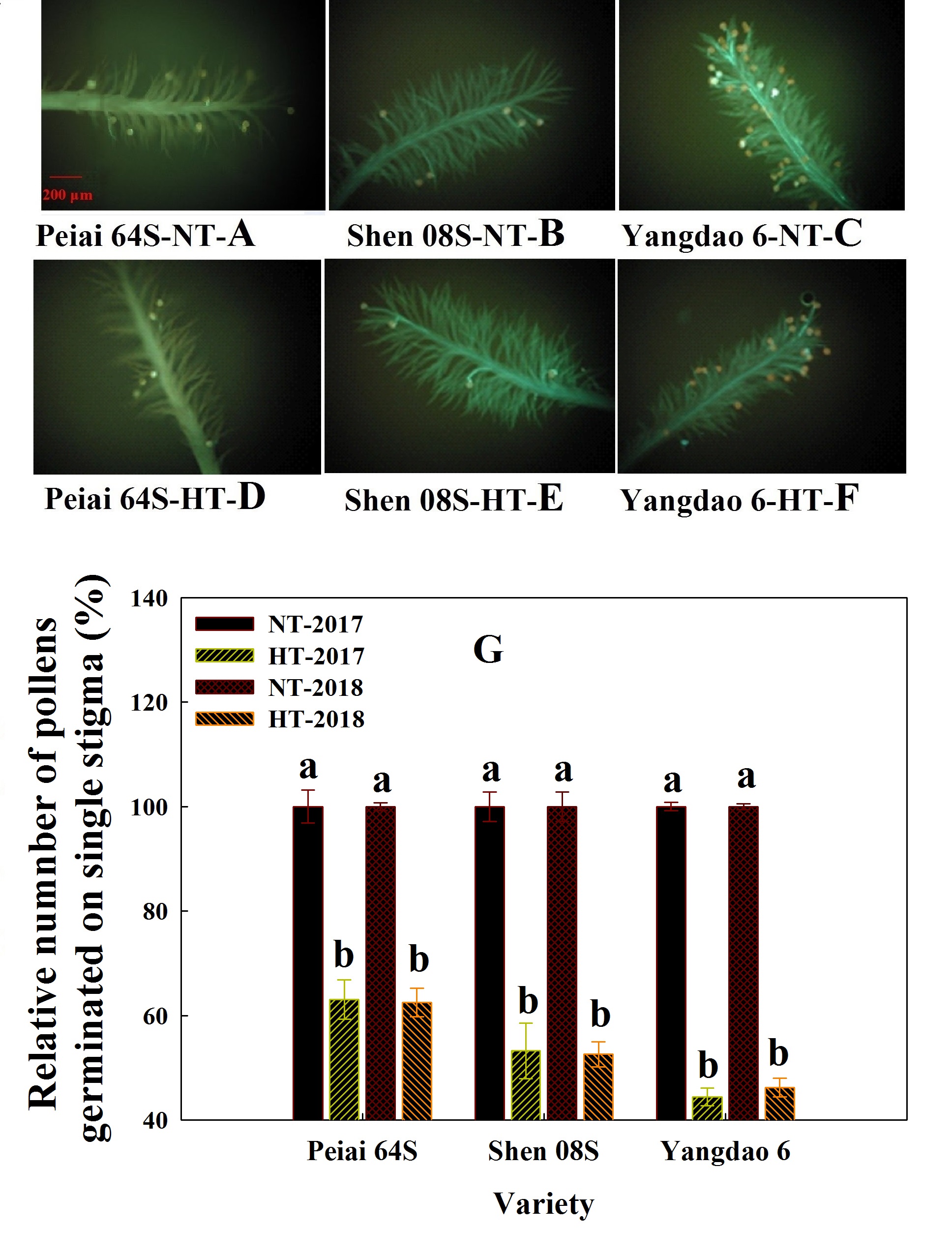

Supplement: Supplementary Figure 2 — Effect of high-temperature stress during anthesis on the number of pollens germinated on stigma of rice. NT and HT represent normal temperature and high temperature, respectively. Luminous circles in (A–F) indicate germinated pollens. Data in G are expressed as relative values, and those of the control (NT) are taken as 100%. The bars in G are SD of six biological replications. Different letters above the bars represent significant differences at the P ≤ 0.05 level within the same line. The images of pollens germinated on single stigma were taken at 50× magnifications. [file Image_2.jpg]
